# Music psychology-based vocal performance anxiety management strategies in pedagogical practice: a mixed-methods intervention study with 3 month follow-up

**DOI:** 10.3389/fpsyg.2026.1752831

**Published:** 2026-04-24

**Authors:** Qin Cong

**Affiliations:** School of Art, Nantong University, Nantong, Jiangsu, China

**Keywords:** heart rate variability (HRV), intervention study, mixed methods, moderating effect, music psychology, psychological resilience, vocal performance anxiety

## Abstract

Vocal performance anxiety is a common psychological barrier among vocal students, severely impacts their skill development, stage performance, and overall wellbeing. This study aims to comprehensively evaluate the short-term and long-term effects of an integrated pedagogical model for anxiety management, constructed based on music psychology theories, through a mixed-methods study with a pre-test, post-test, and 3 month follow-up. We recruited 60 undergraduate vocal performance majors and randomly assigned them to an experimental group (receiving a 12-week “Awareness-Skill-Simulation-Reflection” four-stage integrated pedagogical intervention) and a control group (receiving traditional technical instruction). We collected data on students' state anxiety in performance, vocal performance quality (expert blind ratings), psychological resilience, self-efficacy, and Heart Rate Variability (HRV) at three time points: pre-intervention (T1), post-intervention (T2), and 3 months post-intervention (T3). Additionally, reflective journals from the experimental group were subjected to qualitative analysis. Quantitative findings indicate: (1) Compared to the control group, the integrated intervention significantly reduced the experimental group's vocal performance anxiety and enhanced their performance quality, psychological resilience, and HRV levels. These positive effects remained stable 3 months after the intervention concluded. (2) The intervention's effectiveness was moderated by students' initial trait anxiety levels, meaning the intervention was more potent for students with high trait anxiety. (3) Psychological resilience was identified as a potential statistical mediator of the relationship between the pedagogical intervention and the reduction in anxiety levels. Qualitative results revealed profound subjective experiences among students across four dimensions: “Shifting Mindsets,” “Enhanced Bodily Control,” “Reconstructing the Stage Experience,” and “Trust in Teacher-Student Relationship.” This study provides evidence for the effectiveness and durability of this integrated pedagogical model and explores its potential mechanisms, offering a scientific reference for modern vocal education reform.

## 1 Introduction

Singing, as an ancient and universal form of human emotional and artistic expression, has the core educational goal of cultivating students' exceptional vocal techniques and profound musical understanding. However, on the path to artistic excellence, a long-standing yet often overlooked “hidden obstacle”-vocal performance anxiety—deeply troubles countless vocal students (Barros et al., 2022). Vocal performance anxiety, as the specific manifestation of music performance anxiety (MPA) in the vocal domain, can be understood as a complex emotional response characterized by tension, fear, and worry, accompanied by physiological, cognitive, and behavioral dysregulation, experienced by an individual when facing evaluative performance situations (Kenny, 2011). Its impact extends far beyond a single on-stage error, potentially leading to a decline in learning interest, hindered career development, and even long-term mental health issues (Henshaw and Collyer, 2022). The intimate connection between voice and emotional expression makes vocal performance particularly complex, thereby exacerbating anxiety problems (Correia et al., 2022).

Traditional vocal pedagogy largely follows a technique-centered path: breath control, vocal placement, resonance adjustment, articulation, and enunciation. While this teaching model is undoubtedly crucial for building students' foundational vocal technique, it largely treats singing as a purely physiological-physical process, neglecting the decisive influence of the vocalist's holistic psychological state on vocal production (Shi, 2022). A pedagogical perspective that integrates psychological factors is becoming a significant trend in modern art education strategies (Chen and Dong, 2024). When a student's muscles stiffen and breathing becomes shallow due to inner fear, even the most refined vocal instruction may prove ineffective. As renowned vocal pedagogue Richard Miller stated, singing is a “psycho-physiological” unified activity, where an imbalance in either aspect can disrupt overall harmony. This division between technique and psychology constitutes the primary challenge faced by current vocal education in addressing performance anxiety.

In recent years, with the flourishing development of music psychology, researchers have provided a wealth of theoretical tools for intervening in MPA, such as Cognitive Behavioral Therapy (CBT), mindfulness practices, and exposure therapy (Gómez-López and Sánchez-Cabrero, 2023; Kinney et al., 2025). Specific intervention programs also vary widely, including Acceptance and Commitment Therapy (ACT) practices (Mahony et al., 2022) and intervention projects for choirs (Granados and Bonastre, 2021). However, a clear gap still exists between these valuable theoretical achievements and frontline vocal teaching practice. The psychological mechanisms and developmental roots of music performance anxiety (MPA) have been extensively studied (Kenny, 2011), with early sensitizing experiences considered one of the important influencing factors (Osborne and Kenny, 2008). Yet, these valuable theoretical insights often remain disconnected from practical vocal instruction, and a critical question arises regarding the appropriate role of educators in applying these psychologically-informed techniques.

To address this challenge, this study reframes psychologically-informed interventions within a pedagogical, rather than clinical, context. It is based on the premise that music teachers are uniquely positioned to provide first-line psychological support. Research indicates that students often prefer discussing performance-related psychological issues with their teachers, with whom they have frequent contact and established trust (Williamon and Thompson, 2006). Furthermore, students face significant barriers to seeking professional psychotherapy, including stigma, cost, time constraints, and a lack of therapists specializing in performers' issues (Shaw et al., 2020). Therefore, training teachers to act as performance coaches equipped with psychological skills, rather than as psychotherapists, is an ethically sound and pragmatic approach. This study aims to comprehensively examine the effectiveness, durability, and underlying mechanisms of a systematic framework that translates psychological knowledge into actionable pedagogical practices for vocal teachers. We have constructed a specific, actionable “Awareness-Skill-Simulation-Reflection” four-stage integrated application model, named the “Psychological Scaffolding Model in Vocal Pedagogy.” The core idea of this model is that teachers provide systematic psychological support, building temporary cognitive, physiological, and behavioral “scaffolds” for students to help them ascend to higher artistic and psychological levels. Once students' own “psychological core strength” is robust enough, these scaffolds can be removed.

Based on this, we propose the following four core research hypotheses:

H1 (Effectiveness Hypothesis): Compared to the control group receiving traditional instruction, students in the experimental group undergoing the “Psychological Scaffolding Model” intervention will experience a significant reduction in vocal performance state anxiety and a significant improvement in vocal performance quality, psychological resilience, self-efficacy, and Heart Rate Variability (HRV) post-intervention.

H2 (Durability Hypothesis): The positive effects gained by the experimental group will be maintained 3 months post-intervention, demonstrating good durability.

H3 (Mechanism Hypothesis): We hypothesize that Psychological resilience will emerge as a statistical mediator in the relationship between the pedagogical intervention and the reduction in vocal performance anxiety, reflecting a potential pathway that is consistent with our theoretical model.

H4 (Boundary Condition Hypothesis): The effectiveness of the pedagogical intervention will be moderated by students' initial trait anxiety levels, with more significant effects for students with high trait anxiety.

## 2 Theoretical framework and intervention model design

### 2.1 Multifaceted psychological roots of vocal performance anxiety

This study's intervention model is built upon an understanding of the multidimensional roots of vocal performance anxiety, integrating perspectives from various psychological schools of thought.

Cognitive Psychology Perspective: Unhelpful Thinking Patterns. Cognitive psychology posits that anxiety is triggered not by events themselves, but by an individual's interpretation of those events. Core cognitive characteristics of vocal performance anxiety include: “catastrophizing thoughts” that imagine minor errors as devastating failures; “maladaptive perfectionism” that sets unrealistic standards and evaluates in black-and-white terms; and the voice of an “inner critic” that constantly self-deprecates (Yang et al., 2025).

Physiological Psychology Perspective: The Body's Stress Response. When the brain interprets a performance situation as a “threat,” the autonomic nervous system activates the “fight-or-flight” response, leading to a surge in adrenaline and cortisol. This triggers a cascade of reactions such as increased heart rate, shallow breathing, and muscle tension, which can directly undermine the breath support and fine muscular coordination of the laryngeal, neck, and jaw muscles crucial for phonation (Khazan, 2024).

Neuropsychological Perspective: Neurobiological Correlates of Anxiety. From a neural mechanism standpoint, anxiety involves a dysregulation between the amygdala (involved in threat detection) and the prefrontal cortex (involved in executive control and regulation). In anxious states, heightened amygdala activity can override the regulatory functions of the prefrontal cortex, leading to a state dominated by fear and reactive responses rather than rational thought.

Socio-Environmental Psychology Perspective: The Pressure of Social Evaluation. The evaluative nature of performance makes it a social activity. The threat of judgment from audiences, judges, and teachers, as well as social comparison with peers, are significant external sources of anxiety (Qiu, 2023).

Self-Determination Theory Perspective: Frustrated Psychological Needs. Self-Determination Theory (SDT) posits that when an individual's three basic psychological needs—autonomy, competence, and relatedness—are threatened in a learning environment (e.g., feeling forced to sing, receiving continuous negative feedback, lack of support), it can lead to anxiety and maladaptive coping patterns (Turhal, 2022).

### 2.2 The “psychological scaffolding” integrated intervention system

Based on the theories above and drawing upon established psychological skills training frameworks in sport and performance psychology (Weinberg and Gould, 1999), we constructed an integrated intervention system comprising four strategic modules.

Module One: Cognitive Skill Training. The core goal is to identify, challenge, and modify irrational automatic thoughts that lead to anxiety (Beck, 2020). Teachers guide students to become “detectives” of their own thoughts, learning to replace catastrophizing thoughts (e.g., “I'm definitely going to mess up”) with more rational, adaptive ones (e.g., “This is a challenge, I'll do my best”) through a “Catch-Challenge-Change” three-step process.

Module Two: Physiological Relaxation & Regulation. This aims to directly intervene in the physiological responses of anxiety through conscious bodily training. Key techniques include:

Diaphragmatic Breathing (Abdominal Breathing): Such as the “4-7-8” breathing technique, which can directly activate the parasympathetic nervous system, lower heart rate, and relax muscles.

Progressive Muscle Relaxation (PMR): Through systematically “tensing-relaxing” various muscle groups throughout the body, students learn to perceive and release unconscious bodily tension.

Body Scan: Guiding attention to different parts of the body with a non-judgmental attitude, enhancing the mind-body connection, and detecting tension signals early.

Module Three: Behavioral Skill Training & Graded Performance Practice. This emphasizes reducing anxiety by changing behavioral patterns and systematically confronting feared situations in a supportive pedagogical setting.

Graded Performance Practice: Collaborating with students to create a “performance anxiety hierarchy,” starting from the lowest anxiety situations (e.g., practicing alone) and gradually progressing to higher-level challenges (e.g., singing for friends, performing in a small recital) while maintaining a relaxed state.

Simulated Performance: Rehearsing as much as possible in realistic settings before an actual performance, including using mental imagery techniques (Finch and Oakman, 2022), to reduce the unfamiliarity and uncertainty of the live event.

Module Four: Mindfulness & Flow Experience. This aims to shift students' attention from future worries and self-criticism to the present musical experience itself.

Mindfulness Practice: Guiding students to fully focus on each moment during singing—the flow of breath, the vibration of the vocal cords, the emotion of the music—“observing without judgment” any distracting thoughts.

Flow Induction: Helping students enter a “flow state” of complete immersion and timelessness by ensuring a balance between challenge and skill, setting clear process goals, and providing immediate feedback.

**Figure 1:**
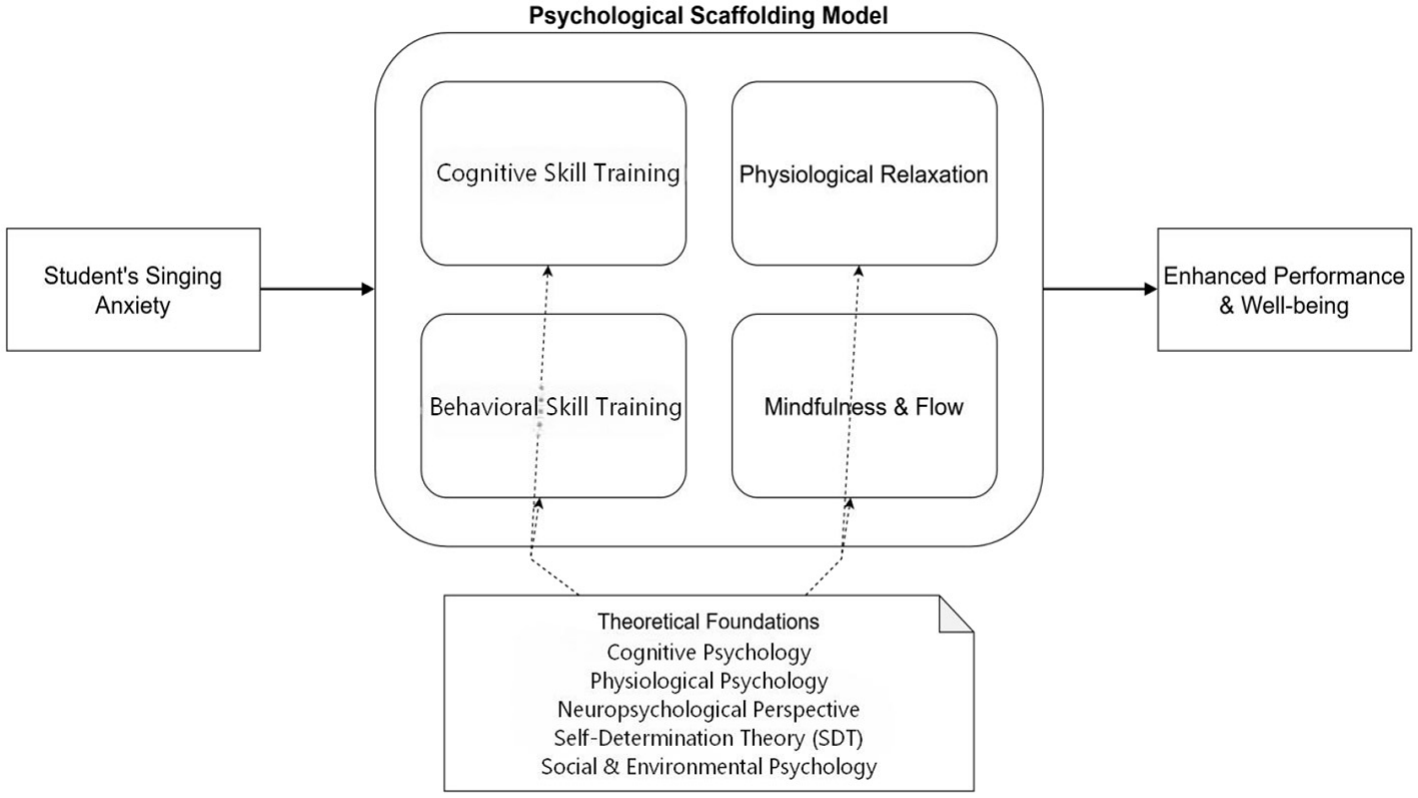
Conceptual framework of the “psychological scaffolding model” in vocal pedagogy.

These four modules are interconnected and progressive, forming a comprehensive intervention system. Derived from diverse theoretical foundations and implemented through specific strategic modules, this system aims to guide students from an initial state of anxiety to ultimately achieving a harmonious integration of artistic expression and wellbeing. Figure 1 visually presents the complete conceptual framework of this model. It is important to note that this figure illustrates the conceptual logic and interconnected components of the intervention design, rather than representing a linear, deterministic causal chain that has been empirically tested.

## 3 Research methods

### 3.1 Research design

This study adopted a pre-test, post-test, control group design with a 3-month follow-up, using mixed methods to comprehensively evaluate the intervention's effects. The study included three time points: pre-intervention (T1), post-intervention (T2), and 3 months post-intervention (T3). At each time point, participants first completed all self-report questionnaires. Following this, they performed their selected vocal piece for recording. The STAI-S was administered approximately 5 min before the performance task began. To minimize order and fatigue effects, the sequence of performance tasks was counterbalanced across participants using a Latin square design. Furthermore, to ensure consistency in performance conditions, all performance recordings were conducted in the same standardized recital hall. The complete administration schedule for each measurement tool at the three time points is detailed in Appendix 1.

### 3.2 Participants

Through campus posters and course recommendations, 80 undergraduate vocal performance majors were recruited from a comprehensive university's music conservatory. After screening (excluding students currently receiving psychological treatment or with a history of severe cardiovascular disease), 60 students (mean age *M*=19.3 years, SD=0.85; 83.3% female) ultimately agreed to participate in the study. They were randomly assigned to the experimental group (n=30) and the control group (n=30) at a 1:1 ratio using a computer-generated random number table.

### 3.3 Intervention procedure

The total intervention duration was 12 weeks, with one 90-min session per week. Both the experimental and control groups were matched on total instructional time and teacher contact. The 4 teachers (2 male, 2 female, with an average of 12.5 years of teaching experience) were all voice professors from the same university. Two teachers were randomly assigned to the experimental group and two to the control group.

To ensure effective intervention delivery, we paid particular attention to intervention fidelity and acceptability (He et al., 2024). Specifically, to ensure fidelity, experimental group teachers received 20 hours of systematic training on the “Psychological Scaffolding Model.” This training was designed and delivered by a licensed clinical psychologist with over 15 years of experience in CBT and performance psychology. The training comprised modules on theoretical foundations (8 h), practical skill delivery (8 h), and protocol monitoring and ethical boundaries, including when to refer students for professional mental health support (4 h). A summary of the teacher training manual is provided in Appendix D.

Throughout the intervention, teachers completed weekly teaching logs to monitor the instructional process. These logs documented core fidelity metrics, primarily content completion rate (verifying all planned topics were covered) and student engagement level (rating participation and attentiveness). The research team reviewed these logs weekly to ensure adherence to the protocol and maintain high intervention fidelity.

Experimental Group: Strictly followed the “Awareness-Skill-Simulation-Reflection” four-stage integrated pedagogical model.

Phase One: Awareness Building (Weeks 1-2). Helped learners recognize the impact of psychological factors (e.g., anxiety, lack of confidence) on performance and begin to perceive their own psychological states and reaction patterns. Key content included popularizing theories of performance anxiety, self-awareness training, and trigger factor identification.

Phase Two: Skill Acquisition (Weeks 3-6). Systematically taught specific psychological skills and strategies to cope with psychological challenges, including relaxation techniques (e.g., 4-7-8 breathing, progressive muscle relaxation), cognitive skill training, attention control, goal setting (SMART principles), and emotion management techniques.

Phase Three: Simulation & Application (Weeks 7-10). Provided learners with a safe, controlled environment to gradually apply and practice learned psychological skills through organizing small simulated performances, graded performance practice exercises, and stress situation drills. For instance, in graded practice, students would apply relaxation skills while performing for a single peer, then for a small group, progressively increasing the challenge in a supportive context.

Phase Four: Reflection & Internalization (Weeks 11-12). Helped learners review and summarize experiences, integrating learned skills with personal experiences to make them part of their personal coping strategies. In this phase, students were required to write reflective journals after each practice or simulated performance, following structured guidelines detailed in Appendix C, documenting bodily sensations, thought processes, coping strategies used, and their effectiveness, to promote deeper internalization of the intervention effects.

Control Group: Received traditional vocal technical instruction, for the same duration and frequency as the experimental group. The focus was on vocalization techniques and repertoire interpretation, without systematically addressing psychological adjustment strategies. While teachers in the control group were instructed to adhere to a traditional technique-focused curriculum, no systematic fidelity checks were conducted to formally assess the extent to which they might have informally addressed students' psychological concerns.

### 3.4 Quantitative measurement tools

To comprehensively assess the intervention's effects, this study utilized the following standardized scales, physiological indicators, and expert rating methods. For the self-report scales (STAI, CD-RISC, GSES), as there were no single, universally adopted Chinese versions suitable for our specific population of vocal performance majors, we employed a rigorous translation and back-translation procedure to ensure conceptual and linguistic equivalence (Brislin, 1970). The original English scales were translated into Mandarin Chinese by a bilingual psychologist. A second, independent bilingual expert, who was blind to the original English versions, then translated the Chinese drafts back into English. The research team compared the back-translated versions with the originals, and minor discrepancies were discussed and resolved to produce the final versions used in the study. The complete content of all questionnaires and scales is provided in Appendix 2.

Vocal Performance State Anxiety: Measured using the State-Trait Anxiety Inventory – State Form (STAI-S; Spielberger et al., 1983). This 20-item scale measures participants' immediate anxiety levels at specific moments (e.g., before or during performance). Responses are on a 4-point Likert scale (1 = “Not at all,” 4 = “Very much so”), with total scores ranging from 20-80; higher scores indicate greater state anxiety. In the current study, this scale demonstrated excellent internal consistency, with a Cronbach's alpha of.91.

Trait Performance Anxiety: Measured using the State-Trait Anxiety Inventory – Trait Form (STAI-T) at baseline (T1) to assess participants' general anxiety disposition, included as a potential moderating variable in the analysis. This 20-item scale uses a 4-point Likert scale (1 = “Almost never,” 4 = “Almost always”), with total scores ranging from 20-80. Its Cronbach's α in this study was.88.

Vocal Performance Quality: Independently blind-rated by three experienced vocal professors (unaware of group assignments) on anonymized performance recordings. Ratings used a unified multi-dimensional rating scale, detailed in Appendix B, covering five dimensions: pitch accuracy, rhythmic accuracy, tone quality and vocal technique, musical expression and emotional engagement, and stage presence and focus. Each dimension was scored on a 1-10 scale, and an overall performance score (1-10) was provided. This scale was chosen as it reflects the core assessment criteria commonly used in university-level vocal pedagogy, thus ensuring ecological validity. All three raters, who were not involved in the intervention, received a 2-h training session to standardize their use of the scale and rating criteria. Their competency was confirmed through rating a set of pilot recordings prior to the study. Inter-rater consistency (ICC) was 0.85, indicating good reliability of the ratings.

Psychological Resilience: Measured using the Connor-Davidson Resilience Scale (CD-RISC; Connor and Davidson, 2003), a 25-item scale assessing an individual's ability to adapt and recover in adversity. Responses are on a 5-point Likert scale (0 = “Not true at all,” 4 = “True nearly all the time”), with total scores ranging from 0-100; higher scores indicate greater psychological resilience. This scale is widely used in stress coping research; its Cronbach's α in this study was 0.92.

Self-Efficacy: Measured using the General Self-Efficacy Scale (GSES; Schwarzer and Jerusalem, 1995), a 10-item scale assessing an individual's confidence in successfully coping with various challenges. Responses are on a 4-point Likert scale (1 = “Not at all true,” 4 = “Completely true”), with total scores ranging from 10-40; higher scores indicate greater self-efficacy. Its Cronbach's α in this study was.87.

Heart Rate Variability (HRV): Electrocardiogram (ECG) signals were collected during performance tasks using a Polar H10 heart rate monitor. The time-domain index SDNN (standard deviation of all normal R-R intervals, in ms) was analyzed using Kubios HRV Premium software, serving as a physiological indicator of overall autonomic nervous system regulatory capacity (Task Force of The European Society of Cardiology and The North American Society of Pacing and Electrophysiology, 1996). Higher SDNN values indicate greater autonomic nervous system regulatory capacity and better individual adaptation to stress.

### 3.5 Qualitative data collection

To gain a deeper understanding of students' subjective experiences and to provide a qualitative illustration of the intervention process, students in the experimental group were required to write reflective journals throughout the 12-week intervention. In total, over 300 journal entries were collected from the 30 participants (averaging approximately 10 entries per student), with a particular focus on journaling during the final phase of the intervention (Reflection & Internalization, Weeks 11-12). Journal writing followed structured guidelines, detailed in Appendix C, prompting students to record:

Situation Review (practice/performance content and most challenging moment)

Bodily Sensations (e.g., increased heart rate, muscle tension, other physiological responses)

Thought Processes (inner dialogue, negative or positive thoughts)

Coping Strategies (whether learned skills were used and specific methods)

Effectiveness Reflection (effectiveness of strategies and new discoveries or insights)

These journals were coded and analyzed using thematic analysis to extract key themes and patterns. The analytic procedure involved several steps to enhance trustworthiness. The unit of analysis was each complete journal entry. The coding process was both deductive, with initial codes derived from the intervention's four modules, and inductive, allowing for new themes to emerge directly from the data. To ensure objectivity and reliability, two independent coders (a PhD candidate in music psychology and a senior vocal teacher with 5 years of qualitative research experience) were invited to code the reflective journals. To mitigate potential bias, both coders were blinded to the study's specific quantitative results and hypotheses during the coding process. A random sample of 20% of the journals (n=60 entries) was selected for inter-coder reliability testing using Cohen's Kappa coefficient. The overall Kappa value was 0.83, indicating excellent inter-coder agreement (Landis and Koch, 1977). Discrepancies in coding (e.g., ambiguous classification of “bodily sensations” vs. “coping strategies”) were resolved through group discussions with a third supervisor (a professor specializing in qualitative psychology) until consensus was reached. All remaining journals were coded by the primary coder, with the second coder reviewing 10% of the entries to ensure consistency. Regular debriefing sessions were held among the research team to discuss emerging themes and interpretations, and the primary coder maintained a reflexive journal to acknowledge potential biases.

### 3.6 Data analysis strategy

SPSS 26.0 was used for quantitative analysis. Independent samples *t*-tests were performed on baseline data. To account for the non-independence of data due to students being nested within teachers, a series of Linear Mixed-Effects Models (LMM) were used to test the effectiveness and durability of the intervention. In these models, “Time,” “Group,” and their interaction were treated as fixed effects, while “Teacher” was included as a random effect. Hayes' PROCESS macro (Model 4) was used for mediation analysis, and (Model 1) for moderation analysis. NVivo 12 software was used for thematic analysis of qualitative data, involving coding and theme extraction.

## 4 Research results

### 4.1 Descriptive statistics and baseline equivalence check

Independent samples *t*-tests on T1 data showed no significant differences between the two groups in age, gender distribution, or any core measurement indicators (all *p* >0.05), indicating successful random assignment and that the two groups were at comparable levels at the start of the study (see Table 1).

**Table 1:** Demographic information and descriptive statistics for all variables at t1 for both groups.

Variable	Experimental Group (*n* = 30)	Control Group (*n* = 30)	*t-*value	*p*-value
Age (years)	M = 19.2, SD = 0.8	M = 19.4, SD = 0.9	−0.89	0.378
Gender (Male/Female)	8/22	9/21	χ^2^ = 0.12	0.731
STAI-T (Trait Anxiety)	M = 45.3, SD = 8.2	M =46.1, SD=7.9	−0.39	0.698
STAI-S (State Anxiety)	M = 50.1, SD = 7.5	M = 49.5, SD = 8.1	0.31	0.757
Vocal Performance (1-10)	M = 7.1, SD = 0.8	M = 7.0, SD = 0.9	0.45	0.654
CD-RISC (Resilience)	M = 68.5, SD = 10.1	M = 67.9, SD = 9.8	0.24	0.811
GSES (Self-Efficacy)	M = 25.6, SD = 4.3	M = 26.0, SD = 4.8	−0.34	0.735
HRV-SDNN (ms)	M = 42.1, SD = 9.8	M = 41.5, SD = 10.3	0.23	0.819

### 4.2 Intervention effectiveness and durability

We conducted Linear Mixed-Effects Models (LMM) for each of the five primary outcome variables (state anxiety, vocal performance, psychological resilience, self-efficacy, and HRV-SDNN), with “Time,” “Group,” and their interaction as fixed effects, and “Teacher” as a random effect to account for data clustering.

The descriptive statistics (means and standard deviations) for all variables at the three time points are presented in Table 2.

**Table 2:** Means and standard deviations for all variables at three time points for both groups.

Variable	Time	Experimental Group (M ±SD)	Control Group (M ±SD)
STAI-S	T1	50.1 ±7.5	49.5 ± 8.1
	T2	38.2 ±6.9	48.8 ± 7.9
	T3	39.0 ±7.1	49.1 ± 8.2
Vocal Performance	T1	7.1 ± 0.8	7.0 ± 0.9
	T2	8.4 ± 0.7	7.2 ± 0.8
	T3	8.3 ± 0.7	7.1 ± 0.9
Psychological Resilience	T1	68.5 ± 10.1	67.9 ± 9.8
	T2	80.3 ± 9.5	69.1 ± 10.2
	T3	79.8 ± 9.8	68.5 ± 10.0
Self-Efficacy	T1	25.6 ± 4.3	26.0 ± 4.8
	T2	32.5 ± 4.1	26.8 ± 4.5
	T3	32.1 ± 4.2	26.5 ± 4.6
HRV-SDNN	T1	42.1 ± 9.8	41.5 ± 10.3
	T2	55.6 ± 11.2	42.3 ± 10.5
	T3	54.9 ± 11.5	41.9 ± 10.1

The results of the Linear Mixed-Effects Models are summarized in Table 3. The “Time × Group” interaction effect was highly significant for all five outcome variables. This indicates that the trends of change over time were significantly different between the experimental and control groups, providing strong evidence for the intervention's effectiveness after accounting for teacher-level clustering.

**Table 3:** Results of linear mixed-effects models for the ‘time × group' interaction on each outcome variable.

Variable	*F*-statistic	Degrees of Freedom (df)	*p*-value
State Anxiety (STAI-S)	F = 18.21	(2, 65.83)	< 0.001
Vocal Performance	F = 15.98	(2, 64.91)	< 0.001
Psychological Resilience (CD-RISC)	F = 13.54	(2, 66.12)	< 0.001
Self-Efficacy (GSES)	F = 14.02	(2, 65.45)	< 0.001
HRV-SDNN	F = 16.89	(2, 64.78)	< 0.001

**Figure 2:**
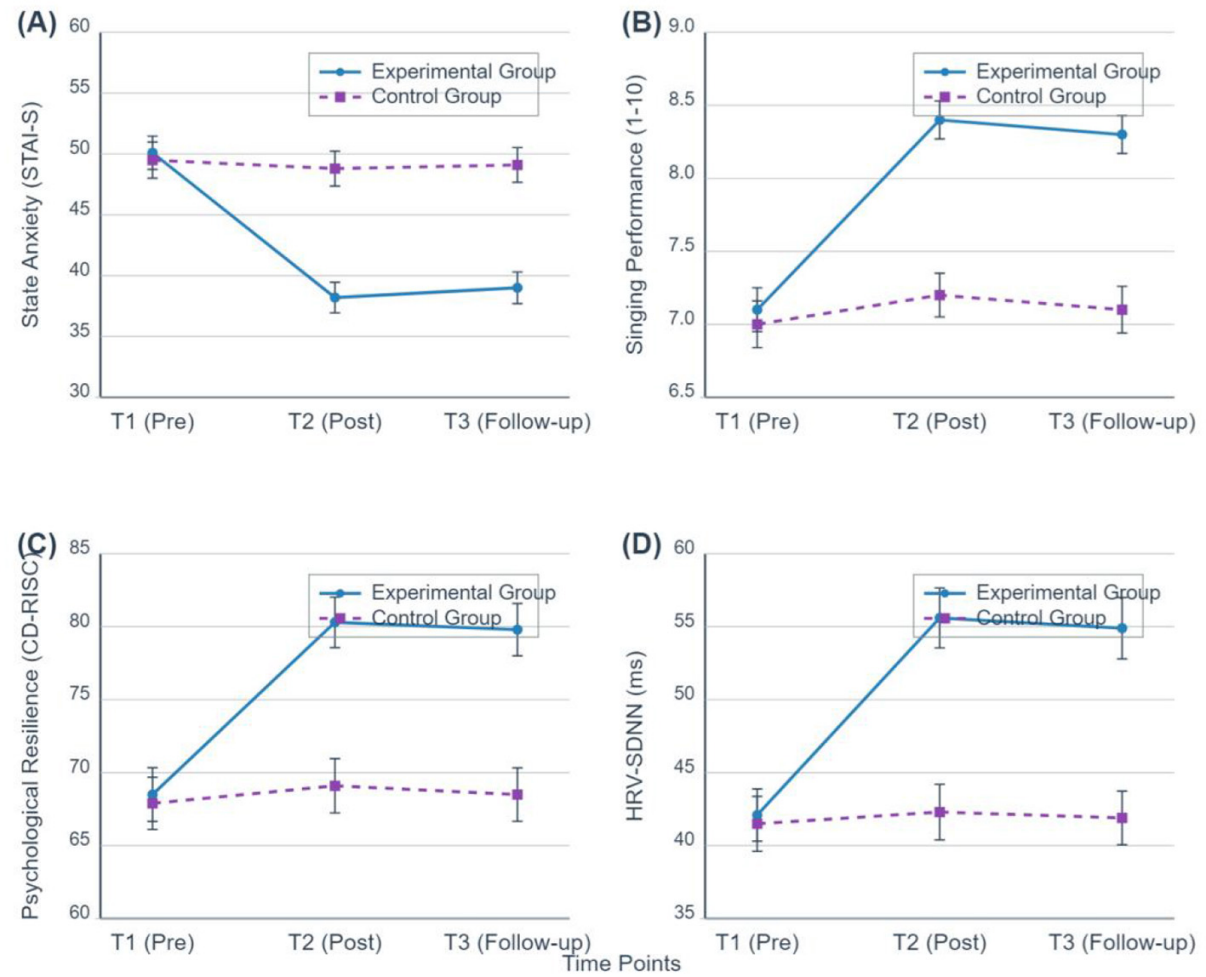
Trends of change in key variables for both groups across three time points. **(A)** State Anxiety; **(B)** Vocal Performance; **(C)** Psychological Resilience; **(D)** Heart Rate Variability. The experimental group is represented by the dark blue solid line (circular markers), and the control group by the purplish-red dashed line (square markers). Error bars represent standard error.

To more clearly illustrate the specific pattern of this interaction, Figure 2 presents the trends of change for four core variables across the three time points for both groups. The figure clearly shows distinct developmental trajectories for the experimental group (solid line) and the control group (dashed line).

*Post-hoc* analyses (with Bonferroni correction) were conducted to examine the specific changes.

Within the experimental group, there was a significant improvement on all outcome measures from pre-intervention (T1) to post-intervention (T2) (all *p* < 0.001), with these gains remaining stable at the 3 month follow-up (T3) (all T2 vs. T3 comparisons, *p* > 0.05). The control group showed no significant changes across any of the time points (all *p* > 0.05).

Between-group comparisons revealed no significant differences at baseline (T1). However, at both post-intervention (T2) and follow-up (T3), the experimental group demonstrated significantly better outcomes on all variables compared to the control group (all *p*<*0.001*).

In summary, these results provide support for our effectiveness hypothesis (H1) and durability hypothesis (H2). The “Psychological Scaffolding Model” integrated intervention not only significantly reduced students' vocal performance anxiety and enhanced their performance quality, psychological resilience, self-efficacy, and autonomic nervous system regulatory capacity in the short term (at the end of the intervention), but more importantly, these positive effects remained stable 3 months post-intervention, demonstrating excellent long-term benefits. This suggests that the changes brought about by this intervention model are not merely transient “Hawthorne effects” or “placebo effects,” but genuinely promote the growth and consolidation of students' intrinsic psychological capabilities.

### 4.3 Mediating role of psychological resilience

To further explore the potential internal mechanisms underlying the intervention's effects and test Hypothesis H3 (that psychological resilience mediates the relationship between intervention and anxiety reduction), we conducted a mediation analysis using Hayes (2017) PROCESS macro (Model 4).

In this model, “Group” was treated as the independent variable (X, reference group = control group = 0, experimental group = 1), post-intervention (T2) “Psychological Resilience Score” as the mediating variable (M), and post-intervention (T2) “State Anxiety Score” as the dependent variable (Y). To control for baseline interference, T1 scores for psychological resilience and state anxiety were included as covariates. The bootstrapping test was performed with 5,000 resamples.

The analysis results are shown in Table 4. After controlling for covariates, the analysis revealed that the pedagogical intervention had a significant positive predictive effect on psychological resilience (Path a: β = 0.68, *p* < 0.001), and psychological resilience, in turn, had a significant negative predictive effect on state anxiety (Path b: β = −0.26, *p* < 0.01).

**Table 4:** Mediation analysis results.

Path	Effect	SE	*t*	*p*	95% CI
Total effect (c)	−0.70	0.11	−6.36	< 0.001	[−0.92, −0.48]
Direct effect (c')	−0.52	0.12	−4.33	< 0.001	[−0.76, −0.28]
Indirect effect (ab)	−0.18	0.05	-	-	[−0.29, −0.08]

“*Effect” refers to standardized coefficients (*β*)*.

The total effect (Path c) of the pedagogical intervention on state anxiety was significant (β = −0.70, *p* < 0.001). After controlling for the mediating effect of psychological resilience, the direct effect (Path c') of the pedagogical intervention on state anxiety remained significant (β = −0.52, *p* < 0.001), but its magnitude was reduced.

Crucially, the bootstrapping test with 5,000 resamples showed a significant indirect effect of psychological resilience. The standardized indirect effect (Path a × b) was −0.18, with a 95% confidence interval of [−0.29, −0.08], which does not include zero. This indicates that psychological resilience played a significant partial mediating role in a statistical sense between the pedagogical intervention and the reduction in vocal performance state anxiety. This indirect effect accounted for 25.7% of the total effect (|−0.18 / −0.70 | × 100%).

**Figure 3:**
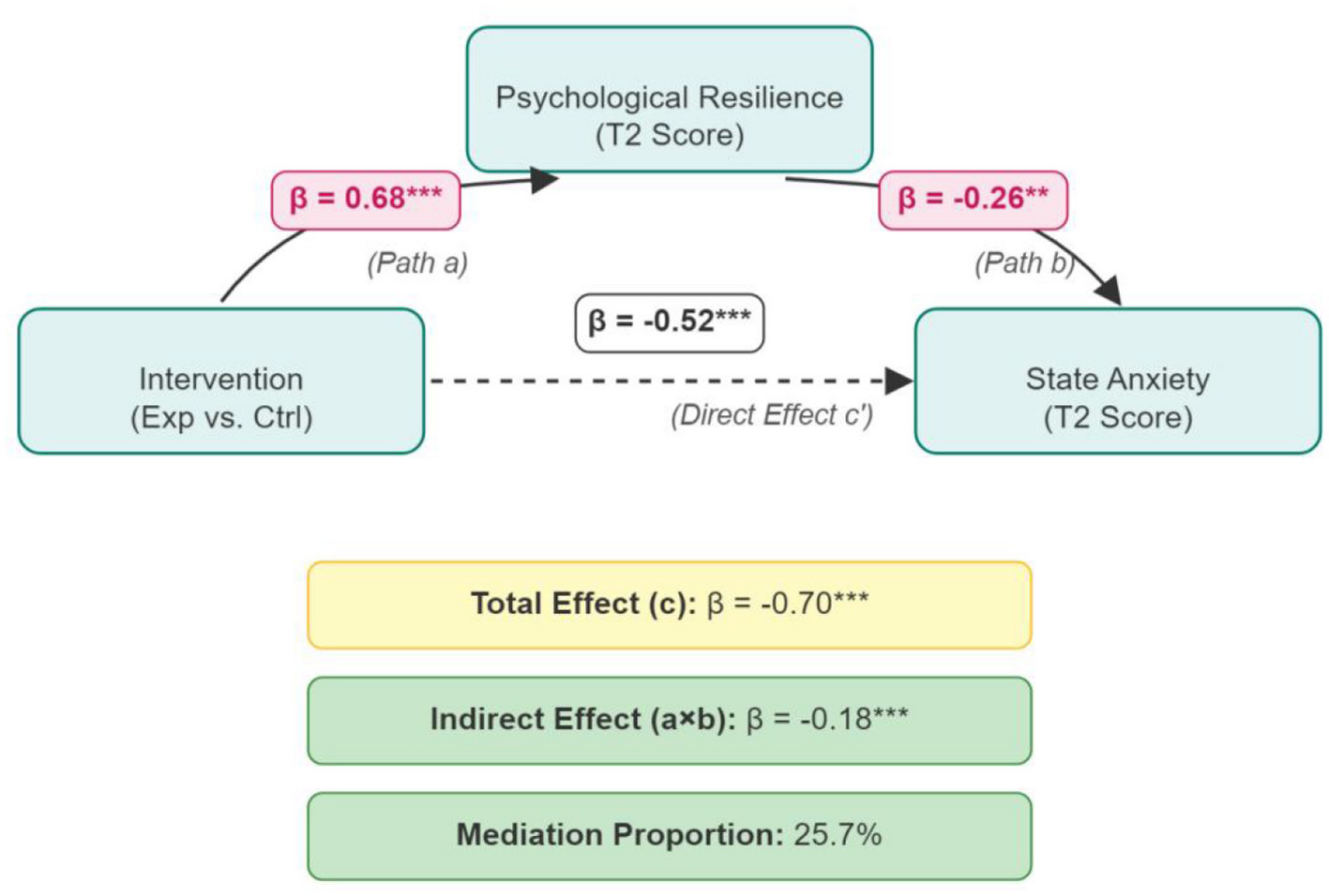
Path diagram of psychological resilience's mediating effect between pedagogical intervention and state anxiety.

Figure 3 clearly illustrates the path relationships and standardized regression coefficients of this mediation effect model.

This result provides support for our hypothesis H3. It suggests a potential internal pathway through which the “Psychological Scaffolding Model” operates: this integrated pedagogical intervention may not only directly address anxiety but also exert its stress-reducing effect by building a stronger “psychological immune system” (i.e., psychological resilience).

However, it is crucial to interpret this finding with caution. Because the mediator (T2 resilience) and outcome (T2 anxiety) were measured concurrently, this analysis cannot establish temporal precedence or rule out unmeasured confounding variables. For instance, it is possible that a reduction in anxiety enhanced feelings of resilience, rather than the other way around. The result should therefore be interpreted as a statistical association consistent with our hypothesis, rather than definitive evidence of a causal psychological mechanism.

### 4.4 Moderating role of initial trait anxiety

To test H4, we used the PROCESS macro (Model 1) to examine the moderating effect of T1 trait anxiety (STAI-T score, mean-centered) on the intervention's effectiveness. Results showed that the “Group × Initial Trait Anxiety” interaction term had a significant negative predictive effect on T2 state anxiety (B = −3.51, p =0.021) (see Table 5).

**Table 5:** Moderation analysis results.

Predictor	*B*	*SE*	*t*	*p*
(Constant)	48.95	0.98	49.95	< 0.001
Group	−11.52	1.85	−6.23	< 0.001
Initial Trait Anxiety (mean-centered)	0.85	0.21	4.05	< 0.001
Group × Initial Trait Anxiety	−3.51	1.50	−2.34	0.021

**Figure 4:**
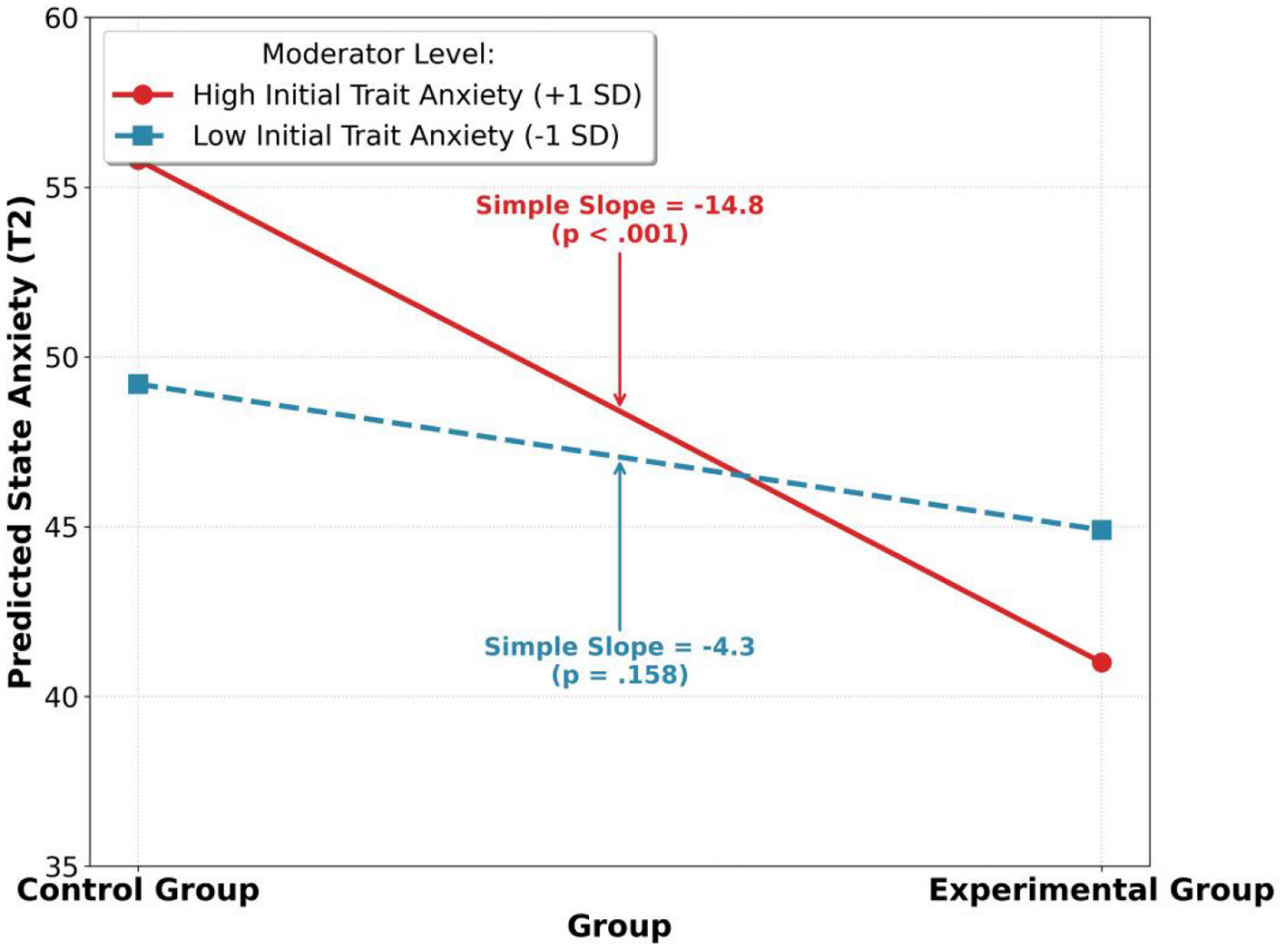
Moderating effect of initial trait anxiety on intervention outcomes.

Simple slope analysis indicated (see Figure 4) that for students with high initial trait anxiety (mean + 1 standard deviation), the experimental group's intervention significantly reduced their state anxiety (b = −14.85, *p* < 0.001); however, for students with low initial trait anxiety (mean-1 standard deviation), the intervention effect was not significant (*b* = −4.32, *p* = 0.158). This confirmed Hypothesis H4, demonstrating that the model is particularly effective for students with high anxiety.

### 4.5 Qualitative analysis findings

To gain a deeper understanding of the “Psychological Scaffolding Model” intervention's impact on students' internal experiences and psychological mechanisms, this study conducted a thematic analysis of over 300 reflective journals submitted by 30 experimental group students throughout the 12-week intervention. Four core themes were ultimately distilled, providing rich context and corroboration for the quantitative data and vividly depicting the specific pathways through which students perceived the intervention effects to have materialized.

Shifting Mindsets: From “Catastrophizing” to a “Growth-Oriented” Perspective. Through what was taught in the cognitive skills module, students reported amending their irrational “catastrophizing” thoughts about performance errors into a more adaptive “growth mindset.” One student noted: “Before, just thinking about that high note terrified me, my mind was full of “I'm definitely going to crack.” Now I tell myself “This is a challenge, try it, and if it cracks, it's okay, it's a learning opportunity.” This reflects a reported shift from rigid self-criticism to self-acceptance.

Enhanced Bodily Control: Breath as an “Emotional Anchor.” Physiological relaxation and regulation training appeared to teach students to view their bodies as effective tools for emotional self-regulation. When faced with high-pressure situations, many reported actively utilizing breathing exercises to manage physiological arousal. One student described: “My heart was pounding while waiting backstage, and I immediately started doing 4-7-8 breathing… it felt like dropping anchor and steadying myself. This feeling of being able to ‘control' nervousness is amazing.” This highlights how students perceived physiological regulation techniques supported psychological security and confidence.

Reconstructing the Stage Experience: From “Judgment Arena” to “Sharing Space.” Students' psychological representation of the “stage” as a performance context seemed to undergo a fundamental transformation. Through behavioral skill training and simulated performances, the stage was reframed from a “judgment arena” filled with threat to a “sharing space” for emotional exchange. One student reflected: “I started shifting my focus from ‘what will they think of me' to ‘what do I want to express with this song.' It felt like I was sharing music, not being judged.”

Trust in Teacher-Student Relationship: From “Authority” to “Ally.” Throughout the intervention, the open, accepting, and supportive environment fostered by the teacher was reported as fundamental for students to dare to expose vulnerability and actively participate. Teachers built deep trust by sharing their own experiences and expressing understanding. A student commented: “What touched me most was when the teacher said, “It's normal to be nervous, I've been nervous too.” This made me feel very safe and more willing to discuss my true thoughts and fears with the teacher…” This shift from a traditional “authority” to an “ally” relationship provided powerful psychological safety support, serving as the cornerstone for the effective implementation of all cognitive, physiological, and behavioral intervention strategies.

In summary, the qualitative analysis findings corroborate the quantitative data, collectively revealing the students' subjective experiences of the “Psychological Scaffolding Model” intervention's effectiveness. It demonstrates that the core of the intervention was perceived by students as helping them, within a trusting and supportive environment, systematically reshape their cognitive patterns, master physiological regulation skills, and redefine performance situations, thereby achieving a profound transition from passively enduring anxiety to actively managing it.

## 5 General discussion

This study rigorously investigated the “Psychological Scaffolding Model's” intervention effects on vocal students' performance anxiety from multiple dimensions using a longitudinal mixed-methods design. The findings not only confirmed the model's short-term effectiveness and long-term durability but also offered insights into its potential internal mechanisms and applicability boundaries, providing important theoretical and practical insights for modern vocal education.

### 5.1 The role and ethical considerations of teachers in anxiety management

A critical consideration of this study is the ethical justification for training teachers, rather than psychotherapists, to deliver psychologically-informed interventions. This study's model is explicitly framed as a pedagogical or coaching intervention, not a clinical one. We argue that this approach is not only ethically sound but also pragmatically necessary. As leading researchers have noted, music students often feel more comfortable discussing performance-related psychological issues with their teachers, with whom they have established relationships (Williamon and Thompson, 2006). Moreover, significant barriers—such as stigma, cost, and the scarcity of specialist therapists—often prevent students from accessing professional mental health care (Shaw et al., 2020). Therefore, equipping teachers with the competencies of a performance coach allows them to ethically integrate psychological skills into their teaching. A crucial part of the teacher training in this study was to define these boundaries, emphasizing that their role is to provide educational support and skills training, not to diagnose or treat clinical disorders. Teachers were also trained to identify signs of severe anxiety that would necessitate referring a student to a qualified mental health professional, ensuring a network of safety for all participants.

### 5.2 Comprehensiveness and objectivity of intervention effects

A core finding of this study is that the integrated intervention demonstrated comprehensive and profound positive impacts. This impact was evident not only in students' self-reported significant decrease in anxiety (subjective psychological level) but also in the significantly improved vocal performance quality as assessed by expert blind ratings (external behavioral level). More importantly, this study innovatively incorporated HRV as an objective physiological indicator. The significant increase in experimental group students' SDNN levels provided strong physiological corroboration for the improvement in psychological state. HRV is a gold standard for measuring autonomic nervous system (ANS) flexibility and adaptability; its increase is typically associated with enhanced parasympathetic activity (i.e., the “relaxation response”) and better emotional regulation capacity (Thayer et al., 2012). This indicates that the “Psychological Scaffolding Model” intervention did not merely superficially alter students' “thoughts” but was associated with genuine optimization of their neurophysiological basis for coping with stress. This tripartite evidence chain (subjective-behavioral-physiological) significantly enhances the credibility of the study's conclusions.

### 5.3 Durability of effects and the internalization mechanism of the “psychological scaffolding”

The follow-up design of this study revealed the excellent durability of the intervention's effects. 3 months post-intervention, the experimental group's advantages across all positive indicators remained robust, suggesting that these changes were not fleeting “Hawthorne effects” but were successfully internalized by students as stable personal capabilities. The statistical mediation analysis provided a preliminary, correlational insight into a potential mechanistic explanation for this: the integrated intervention sustainably reduces anxiety partly because it successfully builds students' “psychological immunity”—psychological resilience. Through cognitive restructuring, mindfulness practices, and other components, the model systematically trained students' psychological resilience, enabling them to more effectively self-regulate and recover quickly when facing future pressures. This perfectly aligns with our initial rationale for naming it the “Psychological Scaffolding Model”: it is not an external crutch requiring lifelong reliance, but a temporary scaffolding that helps students build internal “psychological core strength,” so that once the scaffolding is removed, students can independently and steadily face performance challenges.

### 5.4 Applicability boundaries of the intervention: implications for precision pedagogy

The discovery of the moderating effect offers important guidance for the practical application of this model. Our intervention was particularly effective for students with high initial trait anxiety, suggesting that teachers can use non-clinical, observational methods to identify students who may benefit most. It is crucial to note that clinical assessment tools like the STAI are not recommended for teachers' daily use. Instead, teachers can be trained to look for observable patterns of MPA across four categories: physiological (e.g., trembling, shortness of breath), cognitive (e.g., frequent self-criticism, memory lapses), behavioral (e.g., avoidance of practice), and emotional (e.g., excessive worry, irritability), as recommended by researchers like Shaw et al. (2020). This allows for “precision targeting” and maximizing the efficiency of teaching resources. This finding also aligns with the “Vulnerability-Stress Model,” where interventions provide more significant protective effects for individuals with higher potential vulnerability.

### 5.5 The value of qualitative findings: letting the data “speak”

Qualitative data provided rich insights into the students' subjective experiences, which help to contextualizec the quantitative results. Students' journals suggested that their reported anxiety reduction was connected to replacing “catastrophizing thoughts” with a “growth mindset”; while improved performance and HRV were attributed by them to internalizing techniques like breathing exercises as practical “emotional anchors.” These narratives suggest that the acquisition of these skills was built upon two fundamental transformations: the stage being reinterpreted as a “sharing space” rather than a “judgment arena,” and the teacher-student relationship evolving into a trusting “ally” relationship. This supportive environment created by the teacher, consistent with related research findings (Aydin, 2021; Tahirbegi, 2022), appears to be the cornerstone for students' comprehensive cognitive-to-behavioral transformation. While the majority of narratives were success-oriented, a few journal entries noted difficulties in applying certain cognitive techniques under high pressure, highlighting that the path to skill mastery is not always linear and may require ongoing support.

### 5.6 Limitations and future directions

Despite the rigorous design of this study, several limitations should be acknowledged.

First, a primary limitation is the small number of teachers (four in total) and the design wherein two teachers were nested within each group. Although we employed Linear Mixed-Effects Models to statistically account for data clustering by teacher, which addresses the non-independence of observations, the small number of clusters (i.e., teachers) limits the power and reliability of the random effects estimation. Consequently, we cannot completely rule out the possibility that intervention effects were partially confounded by individual teacher characteristics, such as enthusiasm or specific teaching styles. Future research should aim to replicate these findings with a larger number of teachers to better separate intervention effects from teacher effects.

Second, the sample was drawn from a single university, future research should involve replication across multiple institutions, regions, and even cultural backgrounds to test the model's generalizability. However, it is worth noting that the gender distribution of our sample (approximately 80% female) is consistent with the national average for similar vocal performance programs in China (typically 75-85%), suggesting good representativeness in this demographic aspect.

Third, our control condition was a “business-as-usual” group rather than an active control group. While matched on instructional time, we cannot fully discount that the observed effects in the experimental group were influenced by non-specific factors such as expectancy effects (i.e., the Hawthorne effect), the novelty of the intervention, or simply receiving structured attention and reflection prompts, which were not present in the control group.

Fourth, the intervention in this study was delivered by specially trained teachers, and the extent to which its effects can be generalized to ordinary teachers without systematic training requires further investigation.

Fifth, although it included a 3 month follow-up, longer-term effects (e.g., after 1 year) still need to be explored.

Finally, the concurrent measurement of the mediator and outcome variables in the mediation analysis limits causal interpretation. Future studies could employ a longitudinal design with more time points to establish temporal precedence.

Future research directions could include: (1) exploring the model's applicability and variations for learners of different musical genres (e.g., bel canto, folk, pop, musical theater) and age groups (e.g., adolescents); (2) developing more convenient observational anxiety assessment tools for teachers' daily use; (3) utilizing neuroimaging techniques (e.g., fMRI) to further investigate the long-term effects of the intervention on brain functional networks (e.g., amygdala-prefrontal cortex connectivity); and 4) exploring the feasibility and acceptability of adapting the intervention into online or mobile application formats to reach a wider population of learners (Mirzadegan et al., 2025).

## 6 Conclusion

This study, using a comprehensive array of quantitative and qualitative, subjective and objective, behavioral and physiological indicators, provided substantial evidence for the short-term effectiveness and long-term durability of the “Awareness-Skill-Simulation-Reflection” four-stage integrated pedagogical model (i.e., the “Psychological Scaffolding Model”) in managing vocal students' performance anxiety and enhancing their overall performance and psychological wellbeing. The findings suggested potential mechanisms of action (by enhancing psychological resilience) and its applicability boundaries (more effective for those with high anxiety). This research not only provides a scientifically validated and reproducible practical framework for vocal education reform but also offers new perspectives and strong evidence for how to conduct systematic, effective, and humanistically-oriented mind-body integrated pedagogical interventions in the broader field of performance science.

## Data Availability

The original contributions presented in the study are included in the article/Supplementary material, further inquiries can be directed to the corresponding author.

## References

[B1] Aydin S. (2021). A systematic review of research on teaching anxiety. Int. Online J. Educ. Teach. 8, 730–761.

[B2] Barros S. Marinho H. Borges N. Pereira A. (2022). Characteristics of music performance anxiety among undergraduate music students: a systematic review. Psychol. Music 50, 2021–2043. doi: 10.1177/03057356211066967

[B3] Beck J. S. (2020). Cognitive Behavior Therapy: Basics and Beyond. New York, NY: Guilford Publications.

[B4] Brislin R. W. (1970). Back-translation for cross-cultural research. J. Cross Cult. Psychol. 1, 185–216. doi: 10.1177/135910457000100301

[B5] Chen Y. Dong Z. (2024). Students' psychological analysis for classroom teaching strategies of art songs based on STEAM education. Sustainability 16, 323. doi: 10.3390/su16010323

[B6] Connor K. M. Davidson J. R. (2003). Development of a new resilience scale: the Connor-Davidson resilience scale (CD-RISC). Depress. Anxiety 18, 76–82. doi: 10.1002/da.10113 12964174

[B7] Correia A. I. Castro S. L. MacGregor C. Müllensiefen D. Schellenberg E. G. Lima C. F. (2022). Enhanced recognition of vocal emotions in individuals with naturally good musical abilities. Emotion 22, 894–906. doi: 10.1037/emo0000770 32718172

[B8] Finch K. K. Oakman J. M. (2022). Applied Mental Imagery and Music Performance Anxiety. In Music and Mental Imagery (Thousand Oaks, CA: Routledge), 221–230. doi: 10.4324/9780429330070-24

[B9] Gómez-López B. Sánchez-Cabrero R. (2023). Current trends in music performance anxiety intervention. Behav. Sci. 13:720. doi: 10.3390/bs13090720 37753998 PMC10525579

[B10] Granados L. M. F. Bonastre C. (2021). La ansiedad en la interpretación musical: Programa de intervención en un coro. Rev. Electrón. Compl. Investig. Educ. Musical 18, 49–61. doi: 10.5209/reciem.68541

[B11] Hayes A. F. (2017). Introduction to Mediation, Moderation, and Conditional Process Analysis: A Regression-Based Approach. New York, NY: Guilford publications.

[B12] He S. Shepherd H. Butow P. Shaw J. Harris M. Faris M. . (2024). Fidelity and acceptability of implementation strategies developed for adherence to a clinical pathway for screening, assessment and management of anxiety and depression in adults with cancer. Arch. Public Health 82:65. doi: 10.1186/s13690-024-01293-6 38711115 PMC11071180

[B13] Henshaw A. Collyer S. (2022). Under pressure: reports of performance anxiety across multiple singing genres. J. Sing. 78, 583–590. doi: 10.53830/JETA7812

[B14] Kenny D. T. (2011). The Psychology of Music Performance Anxiety. Oxford: Oxford University Press. doi: 10.1093/acprof:oso/9780199586141.001.0001

[B15] Khazan I. (2024). “Breathing, Heart Rate Variability, and Their Application in Psychotherapy,” in Integrating Psychotherapy and Psychophysiology: Theory, Assessment, and Practice (Thousand Oaks: Routledge), 351–368. doi: 10.1093/oso/9780198888727.003.0016

[B16] Kinney C. Saville P. Heiderscheit A. Himmerich H. (2025). Therapeutic interventions for music performance anxiety: a systematic review and narrative synthesis. Behav. Sci. 15:138. doi: 10.3390/bs15020138 40001769 PMC11851691

[B17] Landis J. R. Koch G. G. (1977). The measurement of observer agreement for categorical data. Biometrics 33, 159–174. doi: 10.2307/2529310 843571

[B18] Mahony S. E. Juncos D. G. Winter D. (2022). Acceptance and commitment coaching for music performance anxiety: Piloting a 6-week group course with undergraduate dance and musical theatre students. Front. Psychol. 13:830230. doi: 10.3389/fpsyg.2022.830230 35369260 PMC8972159

[B19] Mirzadegan I. A. Lewis E. M. Cole S. L. Meyer A. (2025). Perceived acceptability and appropriateness of a web-based program targeting risk for anxiety in young children and their parents. J. Pediatr. Psychol. 50, 6–17. doi: 10.1093/jpepsy/jsae040 38857450 PMC11753869

[B20] Osborne M. S. Kenny D. T. (2008). The role of sensitizing experiences in music performance anxiety in adolescent musicians. Psychol. Music 36, 447–462. doi: 10.1177/0305735607086051

[B21] Qiu Y. (2023). “The evolutionary psychological perspective on social anxiety disorder and its underlying mechanism,” in 2022 4th International Conference on Literature, Art and Human Development (ICLAHD 2022) (Paris: Atlantis Press), 1118–1123. doi: 10.2991/978-2-494069-97-8_142

[B22] Schwarzer R. Jerusalem M. (1995). “Generalized self-efficacy scale,” in Measures in Health Psychology: A User's Portfolio. Causal and Control Beliefs, eds. J. Weinman, S. Wright, and M. Johnston (Windsor: NFER-NELSON), 35–37. doi: 10.1037/t00393-000

[B23] Shaw T. A. Juncos D. G. Winter D. (2020). Piloting a new model for treating music performance anxiety: training a singing teacher to use acceptance and commitment coaching with a student. Front. Psychol. 11:882. doi: 10.3389/fpsyg.2020.00882 32547438 PMC7270208

[B24] Shi H. (2022). Research on the importance of psychological analysis to vocal music singing teaching. Psychiatr. Danub. 34, 510–511.

[B25] Spielberger C. D. Gorsuch R. L. Lushene R. Vagg P. R. Jacobs G. A. (1983). Manual for the State-Trait Anxiety Inventory (Form Y). Palo Alto, CA: Consulting Psychologists Press.

[B26] Tahirbegi D. (2022). Higher music education students' experiences and management of performance anxiety: a qualitative study. Psychol. Music 50, 1184–1196. doi: 10.1177/03057356211034573

[B27] Task Force of The European Society of Cardiology and The North American Society of Pacing and Electrophysiology (1996). Heart rate variability: Standards of measurement, physiological interpretation, and clinical use. Circulation 93, 1043–1065. 8598068

[B28] Thayer J. F. Åhs F. Fredrikson M. Sollers J. J. Wager T. D. (2012). A meta-analysis of heart rate variability and neuroimaging studies: Implications for heart rate variability as a marker of stress and health. Neurosci. Biobehav. Rev. 36, 747–756. doi: 10.1016/j.neubiorev.2011.11.009 22178086

[B29] Turhal E. (2022). Self-regulation behaviors of music education students. Int. J. Res. Educ. Sci. 8, 362–377. doi: 10.46328/ijres.2901

[B30] Weinberg R. S. Gould D. (1999). Foundations of Sport and Exercise Psychology. Human Kinetics.

[B31] Williamon A. Thompson S. (2006). Awareness and incidence of health problems among conservatoire students. Psychol. Music 34, 411–430. doi: 10.1177/0305735606067150

[B32] Yang Y. Lei P. Huang Z. Yu H. Zhang H. (2025). The impact of professional music performance competence on performance anxiety: the mediating role of psychological risk and moderating role of psychological resilience. Front. Psychol. 16:1565215. doi: 10.3389/fpsyg.2025.1565215 40115283 PMC11924045

