# Supplementary material for: Music psychology-based vocal performance anxiety management strategies in pedagogical practice: a mixed-methods intervention study with 3 month follow-up
Source: Front Psychol. 2026 Apr 24;17:1752831. doi: 10.3389/fpsyg.2026.1752831 (PMC13159198; doi:10.3389/fpsyg.2026.1752831)
Supplement: Supplementary file 1 [file Data_Sheet_1.docx]

**Appendix A: Questionnaires and Administration Schedule**

**A.1 Questionnaire Administration Schedule**

This study adopted a longitudinal design, with measurements taken at three time points (T1: pre-intervention, T2: post-intervention, T3: follow-up). The specific administration schedule for each questionnaire tool is shown in the table below:

| Questionnaire Section | T1 (Pre-Intervention) | T2 (Post-Intervention) | T3 (Follow-up) |
| --- | --- | --- | --- |
| 1. Basic Information | √ | × | × |
| 2. Trait Anxiety (STAI-T) | √ | × | × |
| 3. State Anxiety (STAI-S) | √ | √ | √ |
| 4. Psychological Resilience (CD-RISC-25) | √ | √ | √ |
| 5. General Self-Efficacy (GSES-10) | √ | √ | √ |

**A.2 Complete Questionnaire Content**

*The following are all questionnaire items used in this study (presented in the complete T1 questionnaire format):*

**Vocal Learner Psychological State and Performance Research Questionnaire (Phase 1)**

**Dear Student:**

Hello!

We sincerely invite you to participate in an academic study on the psychological state and performance of vocal learners.

Your participation is entirely voluntary, and you may withdraw at any stage of the study without any repercussions. All your responses will be completely anonymous, and the data will be used solely for academic statistical analysis, with strict confidentiality of personal privacy. If you agree to participate, please proceed with the questionnaire; if not, you may close the page. Your continued completion of the questionnaire will be considered as consent to participate in this study.

Thank you for your valuable time and strong support!

## **Research ID (To be filled by researcher):** __________________

**Part I: Basic Information** Please check or fill in your basic information below.

1. **Age:** ______ years old
2. **Gender:** ☐ Male ☐ Female
3. **Major/Specialization:** ☐ Vocal Performance ☐ Music Education ☐ Other (Please specify: __________)
4. **Years of systematic vocal training:** ☐ Less than 1 year ☐ 1-3 years ☐ 3-5 years ☐ More than 5 years

**Part II: General Feelings Survey (STAI-T)** Instructions: Below are some statements that describe how people generally feel. Please read each statement and then check the number on the right that best describes how you generally feel. There are no “right” or “wrong” answers.

| Item | Almost Never | Sometimes | Often | Almost Always |
| --- | --- | --- | --- | --- |
| 1. I feel pleasant. | ☐ 1 | ☐ 2 | ☐ 3 | ☐ 4 |
| 2. I feel nervous and restless. | ☐ 1 | ☐ 2 | ☐ 3 | ☐ 4 |
| 3. I feel satisfied. | ☐ 1 | ☐ 2 | ☐ 3 | ☐ 4 |
| 4. I wish I could be as happy as others. | ☐ 1 | ☐ 2 | ☐ 3 | ☐ 4 |
| 5. I feel like a failure. | ☐ 1 | ☐ 2 | ☐ 3 | ☐ 4 |
| 6. I feel rested. | ☐ 1 | ☐ 2 | ☐ 3 | ☐ 4 |
| 7. I am “calm, cool, and collected.” | ☐ 1 | ☐ 2 | ☐ 3 | ☐ 4 |
| 8. I feel that difficulties are piling up so that I cannot overcome them. | ☐ 1 | ☐ 2 | ☐ 3 | ☐ 4 |
| 9. I worry over unimportant things. | ☐ 1 | ☐ 2 | ☐ 3 | ☐ 4 |
| 10. I feel happy. | ☐ 1 | ☐ 2 | ☐ 3 | ☐ 4 |
| 11. I have disturbing thoughts. | ☐ 1 | ☐ 2 | ☐ 3 | ☐ 4 |
| 12. I lack self-confidence. | ☐ 1 | ☐ 2 | ☐ 3 | ☐ 4 |
| 13. I feel secure. | ☐ 1 | ☐ 2 | ☐ 3 | ☐ 4 |
| 14. I make decisions easily. | ☐ 1 | ☐ 2 | ☐ 3 | ☐ 4 |
| 15. I feel inadequate. | ☐ 1 | ☐ 2 | ☐ 3 | ☐ 4 |
| 16. I am contented. | ☐ 1 | ☐ 2 | ☐ 3 | ☐ 4 |
| 17. Some unimportant thoughts run through my mind and bother me. | ☐ 1 | ☐ 2 | ☐ 3 | ☐ 4 |
| 18. I take disappointments so keenly that I cannot put them out of my mind. | ☐ 1 | ☐ 2 | ☐ 3 | ☐ 4 |
| 19. I am a steady person. | ☐ 1 | ☐ 2 | ☐ 3 | ☐ 4 |
| 20. I get tense and worked up when I think about my present concerns. | ☐ 1 | ☐ 2 | ☐ 3 | ☐ 4 |

**Part III: Immediate Feelings Survey (STAI-S)** Instructions: Below are some statements that describe how people feel *right now, at this moment*. Please read each statement and then check the number on the right that best describes how you feel *right now, at this instant*.

| Item | Not at all | A little | Moderately | Very much so |
| --- | --- | --- | --- | --- |
| 1. I feel calm. | ☐ 1 | ☐ 2 | ☐ 3 | ☐ 4 |
| 2. I feel secure. | ☐ 1 | ☐ 2 | ☐ 3 | ☐ 4 |
| 3. I am tense. | ☐ 1 | ☐ 2 | ☐ 3 | ☐ 4 |
| 4. I am regretful. | ☐ 1 | ☐ 2 | ☐ 3 | ☐ 4 |
| 5. I am at ease. | ☐ 1 | ☐ 2 | ☐ 3 | ☐ 4 |
| 6. I feel upset. | ☐ 1 | ☐ 2 | ☐ 3 | ☐ 4 |
| 7. I am worrying about possible misfortunes. | ☐ 1 | ☐ 2 | ☐ 3 | ☐ 4 |
| 8. I feel satisfied. | ☐ 1 | ☐ 2 | ☐ 3 | ☐ 4 |
| 9. I feel frightened. | ☐ 1 | ☐ 2 | ☐ 3 | ☐ 4 |
| 10. I feel comfortable. | ☐ 1 | ☐ 2 | ☐ 3 | ☐ 4 |
| 11. I feel self-confident. | ☐ 1 | ☐ 2 | ☐ 3 | ☐ 4 |
| 12. I feel nervous. | ☐ 1 | ☐ 2 | ☐ 3 | ☐ 4 |
| 13. I am jittery. | ☐ 1 | ☐ 2 | ☐ 3 | ☐ 4 |
| 14. I feel indecisive. | ☐ 1 | ☐ 2 | ☐ 3 | ☐ 4 |
| 15. I am relaxed. | ☐ 1 | ☐ 2 | ☐ 3 | ☐ 4 |
| 16. I feel content. | ☐ 1 | ☐ 2 | ☐ 3 | ☐ 4 |
| 17. I am worried. | ☐ 1 | ☐ 2 | ☐ 3 | ☐ 4 |
| 18. I feel confused. | ☐ 1 | ☐ 2 | ☐ 3 | ☐ 4 |
| 19. I feel steady. | ☐ 1 | ☐ 2 | ☐ 3 | ☐ 4 |
| 20. I feel pleasant. | ☐ 1 | ☐ 2 | ☐ 3 | ☐ 4 |

**Part IV: Personal Ability and Experience Survey (CD-RISC-25)** Instructions: Please rate the extent to which the following statements describe you over the past month.

| Item | Not true at all | Rarely true | Sometimes true | Often true | True nearly all the time |
| --- | --- | --- | --- | --- | --- |
| 1. I am able to adapt to change. | ☐ 0 | ☐ 1 | ☐ 2 | ☐ 3 | ☐ 4 |
| 2. I can deal with whatever comes my way. | ☐ 0 | ☐ 1 | ☐ 2 | ☐ 3 | ☐ 4 |
| 3. I like challenges. | ☐ 0 | ☐ 1 | ☐ 2 | ☐ 3 | ☐ 4 |
| 4. I can work under pressure. | ☐ 0 | ☐ 1 | ☐ 2 | ☐ 3 | ☐ 4 |
| 5. I tend to bounce back after illness or hardship. | ☐ 0 | ☐ 1 | ☐ 2 | ☐ 3 | ☐ 4 |
| 6. I consider myself a strong person. | ☐ 0 | ☐ 1 | ☐ 2 | ☐ 3 | ☐ 4 |
| 7. I can handle unpleasant feelings. | ☐ 0 | ☐ 1 | ☐ 2 | ☐ 3 | ☐ 4 |
| 8. I can deal with unpleasant or painful feelings, such as sadness, fear, and anger. | ☐ 0 | ☐ 1 | ☐ 2 | ☐ 3 | ☐ 4 |
| 9. When I need to perform well, I put in my best effort. | ☐ 0 | ☐ 1 | ☐ 2 | ☐ 3 | ☐ 4 |
| 10. I can stay focused under pressure. | ☐ 0 | ☐ 1 | ☐ 2 | ☐ 3 | ☐ 4 |
| 11. I am not easily discouraged by failure. | ☐ 0 | ☐ 1 | ☐ 2 | ☐ 3 | ☐ 4 |
| 12. I don’t think I am a person who gives up easily. | ☐ 0 | ☐ 1 | ☐ 2 | ☐ 3 | ☐ 4 |
| 13. I see the humorous side of things when I am faced with difficulties. | ☐ 0 | ☐ 1 | ☐ 2 | ☐ 3 | ☐ 4 |
| 14. Dealing with pressure makes me stronger. | ☐ 0 | ☐ 1 | ☐ 2 | ☐ 3 | ☐ 4 |
| 15. I have a strong will to achieve my goals. | ☐ 0 | ☐ 1 | ☐ 2 | ☐ 3 | ☐ 4 |
| 16. I am proud of my achievements. | ☐ 0 | ☐ 1 | ☐ 2 | ☐ 3 | ☐ 4 |
| 17. I know where to seek help. | ☐ 0 | ☐ 1 | ☐ 2 | ☐ 3 | ☐ 4 |
| 18. I have close and secure relationships. | ☐ 0 | ☐ 1 | ☐ 2 | ☐ 3 | ☐ 4 |
| 19. I can control my life. | ☐ 0 | ☐ 1 | ☐ 2 | ☐ 3 | ☐ 4 |
| 20. I have a sense of control. | ☐ 0 | ☐ 1 | ☐ 2 | ☐ 3 | ☐ 4 |
| 21. I like to take charge of things. | ☐ 0 | ☐ 1 | ☐ 2 | ☐ 3 | ☐ 4 |
| 22. Sometimes I force myself to do things I don’t want to do. | ☐ 0 | ☐ 1 | ☐ 2 | ☐ 3 | ☐ 4 |
| 23. My beliefs give me strength. | ☐ 0 | ☐ 1 | ☐ 2 | ☐ 3 | ☐ 4 |
| 24. Sometimes I have to rely on others. | ☐ 0 | ☐ 1 | ☐ 2 | ☐ 3 | ☐ 4 |
| 25. Sometimes I am lucky or rely on chance. | ☐ 0 | ☐ 1 | ☐ 2 | ☐ 3 | ☐ 4 |

**Part V: Confidence Survey (GSES-10)** Instructions: Please rate the extent to which the following statements describe you.

| Item | Not at all true | Hardly true | Moderately true | Exactly true |
| --- | --- | --- | --- | --- |
| 1. If I try hard enough, I can always solve difficult problems. | ☐ 1 | ☐ 2 | ☐ 3 | ☐ 4 |
| 2. Even when others oppose me, I can find a way to get what I want. | ☐ 1 | ☐ 2 | ☐ 3 | ☐ 4 |
| 3. It is easy for me to stick to my aims and to achieve my goals. | ☐ 1 | ☐ 2 | ☐ 3 | ☐ 4 |
| 4. I am confident that I could effectively deal with unexpected events. | ☐ 1 | ☐ 2 | ☐ 3 | ☐ 4 |
| 5. Thanks to my resourcefulness, I know how to handle unforeseen situations. | ☐ 1 | ☐ 2 | ☐ 3 | ☐ 4 |
| 6. When I am confronted with a problem, I can usually find several solutions. | ☐ 1 | ☐ 2 | ☐ 3 | ☐ 4 |
| 7. I can handle whatever comes my way. | ☐ 1 | ☐ 2 | ☐ 3 | ☐ 4 |
| 8. When I’m in a difficult situation, I can usually think of something to do. | ☐ 1 | ☐ 2 | ☐ 3 | ☐ 4 |
| 9. I can remain calm when facing difficulties because I rely on my coping abilities. | ☐ 1 | ☐ 2 | ☐ 3 | ☐ 4 |
| 10. When I’m confronted with a problem, I can usually find several solutions. | ☐ 1 | ☐ 2 | ☐ 3 | ☐ 4 |

**Appendix B: Vocal Performance Rating Scale (For Expert Evaluation)**

**Reviewer ID:** __________ **Participant Research ID:** __________ **Rating Time Point:** ☐ T1 ☐ T2 ☐ T3

**Instructions:** Please rate the participant’s live performance (or audio/video recording) across the following dimensions and provide an overall evaluation.

| Rating Dimension | Score (1-10) | Descriptive Anchors |
| --- | --- | --- |
| 1. Pitch Accuracy | __________ | 1: Severely off-pitch, 5: Occasional errors, 10: Highly accurate |
| 2. Rhythmic Accuracy | __________ | 1: Severely deviates, 5: Occasionally unsteady, 10: Rhythmical, steady, and accurate |
| 3. Tone Quality & Vocal Technique | __________ | 1: Dry tone/unsteady breath, 5: Fairly stable but lacks color, 10: Rich tone/excellent breath support |
| 4. Musical Expression & Emotional Engagement | __________ | 1: Mechanical/flat, 5: Some interpretation but not fully engaged, 10: Sincere emotion/highly expressive |
| 5. Stage Presence & Focus | __________ | 1: Tense/distracted, 5: Generally appropriate but slightly stiff, 10: Confident/composed/focused |
| Overall Performance Score | __________ / 10 | (Provided by reviewer, aggregating above items) |

**Qualitative Comments:**

(Please briefly describe the participant’s strengths, main issues, and other observations)

**Appendix C: Reflective Journal Writing Guidelines (For Experimental Group)**

**Instructions:**

After each practice or simulated performance, please spend 10-15 minutes recording your honest experiences and feelings as detailed as possible, following the prompts below. This is not an assignment; there are no right or wrong answers, so please record honestly. These records will help you better understand yourself and are an important part of our research.

Please consider and record the following core questions:

1. **Situation Review:**

What exactly did you practice/perform today? (e.g., which section of which song)

What was the most challenging or anxiety-inducing moment throughout the process?

1. **Bodily Sensations:**

In that challenging/anxious moment, what did your body feel like? (e.g., increased heart rate, sweaty palms, rapid breathing, muscle tension, stomach discomfort, dry mouth, etc.)

1. **Thought Processes:**

What were you thinking at that moment? What specific thoughts or “inner dialogue” did you have? (e.g., “I’m definitely going to crack this note,” “The audience will think I’m terrible,” “I forgot the lyrics again, how awful,” etc.)

1. **Coping Strategies:**

Did you try to use any **methods** learned in class to cope with these bodily sensations or thoughts? (e.g., Did you do 4-7-8 breathing? Did you try to challenge negative thoughts in your mind? Did you refocus your attention on the music?)

1. **Effectiveness Reflection:**

If you tried certain methods, did they work? What changes occurred in your bodily sensations or thoughts?

If you didn’t try, what do you think held you back?

What new discoveries or insights did you gain from today’s experience?
