# Supplementary material for: Music psychology-based vocal performance anxiety management strategies in pedagogical practice: a mixed-methods intervention study with 3 month follow-up
Source: Front Psychol. 2026 Apr 24;17:1752831. doi: 10.3389/fpsyg.2026.1752831 (PMC13159198; doi:10.3389/fpsyg.2026.1752831)
Supplement: Supplementary file 2 [file Data_Sheet_2.docx]

**Appendix D: Summary of the 20-Hour Teacher Training Program for the “Psychological Scaffolding Model”**

**Program Goal:** The primary objective of this training program was to equip vocal teachers with the necessary pedagogical skills to implement the “Psychological Scaffolding Model.” The training explicitly emphasized the role of the teacher as a **performance coach**, not a clinical therapist, focusing on educational support and skill-building within a pedagogical context.

**Trainer:** The training was designed and delivered by a licensed clinical psychologist with over 15 years of experience in Cognitive Behavioral Therapy (CBT) and performance psychology.

**Total Duration:** 20 hours

**Module 1: Theoretical Foundations of Performance Anxiety (8 hours)**

**Session 1: Understanding Vocal Performance Anxiety (2 hours)**

Definition, prevalence, and multidimensional impact of MPA on students.

Distinguishing normal performance nerves from debilitating anxiety.

The “Psycho-physiological” nature of singing.

**Session 2: The Anxious Mind - Cognitive Models (2 hours)**

Introduction to cognitive models (e.g., Beck’s cognitive triad, ABC model).

Identifying common unhelpful thinking patterns in performers (e.g., catastrophizing, black-and-white thinking, perfectionism).

**Session 3: The Anxious Body - Physiological & Neurobiological Basis (2 hours)**

The autonomic nervous system (ANS): “Fight-or-Flight” vs. “Rest-and-Digest.”

The role of Heart Rate Variability (HRV) as an indicator of self-regulation.

Understanding the neurobiology of anxiety in simple terms (e.g., the interplay between the amygdala and prefrontal cortex).

**Session 4: The Performance Context - Social & Motivational Factors (2 hours)**

The impact of social evaluation and audience pressure.

Self-Determination Theory (SDT): Fostering autonomy, competence, and relatedness in the studio to build intrinsic motivation and reduce anxiety.

**Module 2: Practical Skill Delivery and Coaching Techniques (8 hours)**

**Session 5: Coaching Cognitive Skills (2 hours)**

Hands-on training in the “Catch-it, Challenge-it, Change-it” technique.

Role-playing scenarios: Guiding a student to reframe negative self-talk into constructive, realistic thoughts.

**Session 6: Coaching Physiological Regulation (2 hours)**

Instruction and practice: Leading guided diaphragmatic breathing (e.g., 4-7-8 technique), Progressive Muscle Relaxation (PMR), and Body Scan meditations.

How to integrate these techniques into a vocal warm-up routine.

**Session 7: Coaching Behavioral Skills & Graded Performance Practice (2 hours)**

How to collaboratively create a “performance practice hierarchy” with a student.

Structuring and facilitating safe, simulated performance environments.

Using mental imagery and rehearsal techniques.

**Session 8: Fostering Mindfulness and Flow (2 hours)**

Introducing core concepts of mindfulness (“non-judgmental present-moment awareness”).

Strategies to help students focus on the musical process rather than the outcome to facilitate a “flow state.”

**Module 3: Protocol Monitoring and Ethical Boundaries (4 hours)**

**Session 9: The Teacher’s Role: Coach vs. Therapist (2 hours)**

**Defining Boundaries**: Explicit discussion on the scope of practice. What teachers can and should do (coach skills, provide support) vs. what they must not do (diagnose, treat mental disorders).

**Identifying Red Flags**: Training to recognize signs of severe depression, clinical anxiety disorders, or other mental health crises that require professional intervention.

**Referral Procedures**: Establishing a clear, simple protocol for referring students to the university’s counseling and psychological services.

**Session 10: Intervention Fidelity and Building Trust (2 hours)**

Review of the four-stage model and how to adhere to the protocol.

Using the weekly teaching logs for monitoring.

The importance of confidentiality, empathy, and creating a psychologically safe teacher-student relationship as the foundation for the model’s success.
